# The learning curve of endoscopic total mastectomy in Taiwan: A multi-center study

**DOI:** 10.1371/journal.pone.0178251

**Published:** 2017-06-08

**Authors:** Chin-Sheng Hung, Sheng-Wei Chang, Li-Min Liao, Cheng-Chiao Huang, Shih-Hsin Tu, Shou-Tung Chen, Dar-Ren Chen, Shou-Jen Kuo, Hung-Wen Lai, Ting-Mao Chou, Yao-Lung Kuo

**Affiliations:** 1 Division of Breast Surgery, Department of Surgery, Taipei Medical University Hospital, Taipei, Taiwan; 2 Department of Surgery, School of Medicine, College of Medicine, Taipei Medical University, Taipei, Taiwan; 3 Endoscopy & Oncoplastic Breast Surgery Center, Changhua Christian Hospital, Changhua, Taiwan; 4 Division of General Surgery, Changhua Christian Hospital, Changhua, Taiwan; 5 Comprehensive Breast Cancer Center, Department of Surgery, Changhua Christian Hospital, Changhua, Taiwan; 6 School of Medicine, National Yang Ming University, Taipei, Taiwan; 7 Division of Plastic Surgery, Department of Surgery, E-Da Hospital, Kaohsiung, Taiwan; 8 Department of Surgery, National Cheng Kung University Hospital, Tainan, Taiwan; 9 Department of Surgery, College of Medicine, National Cheng Kung University, Tainan, Taiwan; Fu Jen Catholic University, TAIWAN

## Abstract

**Introduction:**

Laparoscopic techniques are commonly used in abdominal and gynecologic surgery, while breast cancer surgery has remained largely unchanged. In Asia, especially in Japan, many surgeons have started to use endoscopic surgery for breast cancer. In Taiwan, endoscopy-assisted breast surgery started in 2010. The benefits of this surgical method include smaller incisions, an axillary anatomic approach, clear vision, no oncologic compromise, and good cosmetic outcomes. This is the first report to discuss the learning curve of endoscopy-assisted breast surgery, including the difficulties experienced.

**Materials and methods:**

From June 2011 to December 2013, data were collected from 134 patients who received an endoscopic total mastectomy at the Taipei Medical University Hospital (TMUH) or Changhua Christian Hospital (CCH). We divided these patients into a learning group (TMUH, n = 15; CCH, n = 15) and a mature group (TMUH, n = 50; CCH, n = 54). Patient data and perioperative variables were recorded by retrospective chart review. Variables were compared using the χ^2^ test and Student’s *t*-test.

**Results:**

There was a significant difference in operation time (275.3 vs. 228.9 minutes, p < 0.01) between the learning and mature groups. Perioperative variables (lymph node dissection method, nipple preservation, and reconstruction method) were also analyzed, but there were no demographic differences between the groups. The complication rate was higher in the learning group, although this difference was also not statistically significant.

**Conclusion:**

Our study is the first to discuss the learning curve of endoscopic total mastectomy. The operation time decreased significantly after 15 cases at each hospital. Although the operation is still more time-consuming than traditional methods, it has the benefit of smaller wounds and improved cosmetic outcomes if combined with immediate reconstruction.

## Introduction

**Fig 1:**
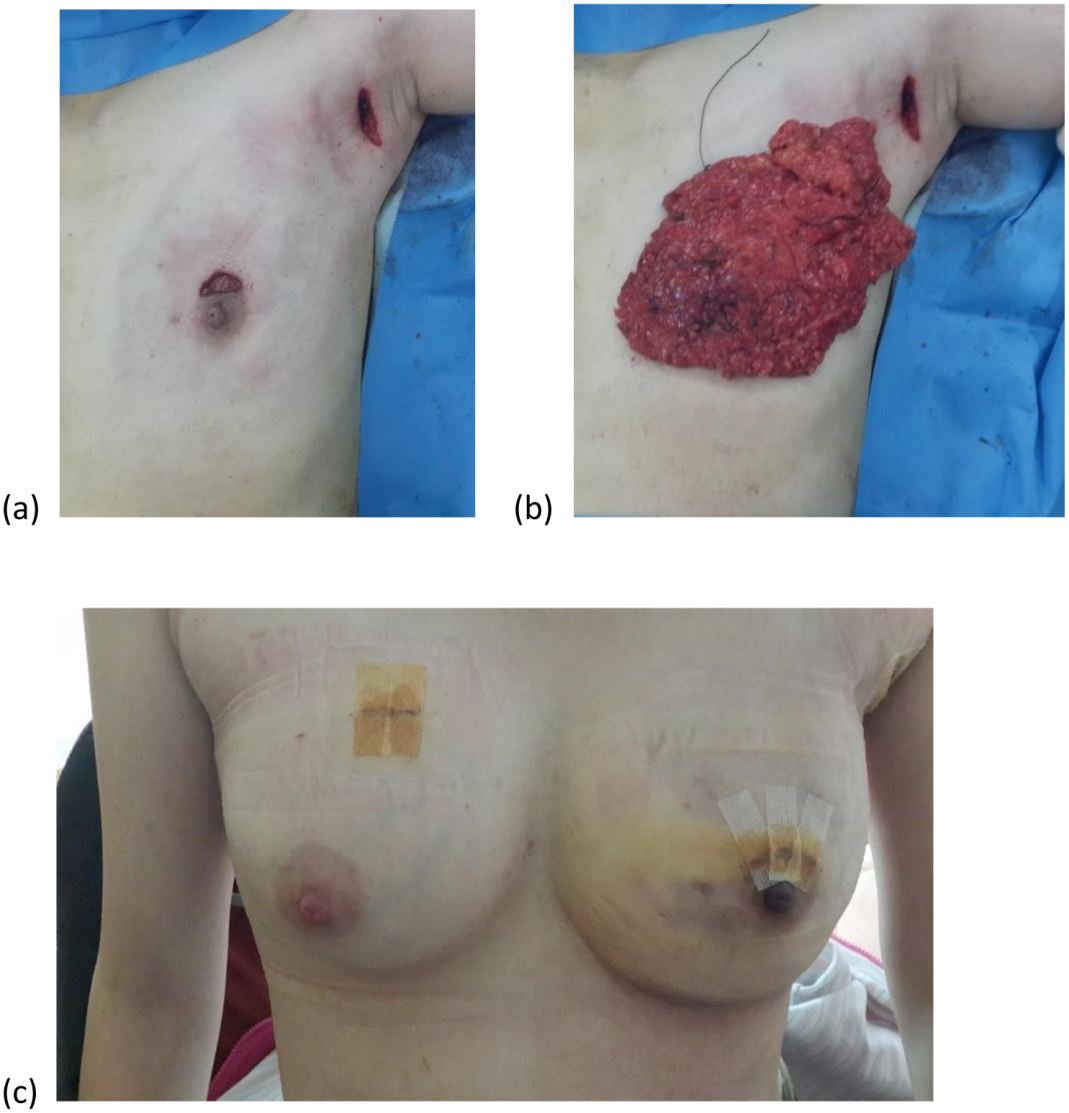
Endoscopic mastectomy. (a) periareolar and axillary incision; (b) the dissected breast tissue; (c) immediate reconstruction with jelly implant (right side is another biopsy for benign fibroadenoma). It has the benefits of smaller incisions, no oncologic compromise, and good cosmetic outcomes.

Breast cancer surgery has been occurring for over a century, but surgical methods have changed little over this time. In recent years, many other types of surgeries have entered the minimally invasive era, e.g. gynecologic and abdominal surgery. Laparoscopic surgery has become a standard and routine procedure in operations such as cholecystectomy or appendectomy. However, the use of the endoscopic method in breast surgery was only proposed in the late 1990s. Many surgeons in Asia have started to use this approach for breast cancer, especially in Japan, with several small series of studies reporting its use for breast tumor excision or mastectomy [1–4]. Most of these studies showed that endoscopic mastectomy is not inferior to traditional surgery [5]. When applied to breast cancer surgery, our previous study found that this surgical method has the benefits of smaller incisions, an axillary anatomic approach, clear vision, no oncologic compromise, and good cosmetic outcomes [6] (Fig 1). While endoscopic surgery has many benefits, this method is not popular among most breast surgeons. Some think it takes more time and is more complex than traditional surgery, due to technical difficulties, as well as instrumentation. However, no previous reports have specifically analyzed endoscopic surgery. We therefore suggest that the learning curve may be the primary obstacle in moving from traditional to endoscopic methods in breast surgery.

The endoscopic surgical method was introduced in Taiwan in 2011, and nearly 300 breast cancer patients have received this type of surgery in the past three years. Three major hospitals—the Taipei Medical University Hospital (TMUH), Changhua Christian Hospital (CCH), and the National Cheng Kung University Hospital (NCKUH)—have the facilities and expertise to perform this operation. We collected patients’ data from TMUH and CCH for retrospective analyses. Our study is the first to investigate the learning curve that occurs when initially performing endoscopic mastectomies. This report is important and useful for breast surgeons intending to introduce this surgical method into their hospitals.

## Materials and methods

From June 2011 to December 2013, data was collected from 203 patients that received this innovative surgical method at TMUH and CCH. The operations were performed by two surgeons, Dr. Hung at TMUH and Dr. Lai at CCH, who were taught the technique by Dr. Fukuma (Kameda Medical Center, Japan). Patients were regularly followed-up every 3 months in the first two years after surgery and then every 6 months until 5 years. In total, 96 patients from TMUH and 107 patients from CCH received an endoscopic mastectomy during this period. These patients were recruited during April 2015 to March 2016 and their written informed consent for data collection was obtained during follow-up. The detail study protocol was described in a previous article [6].

**Fig 2:**
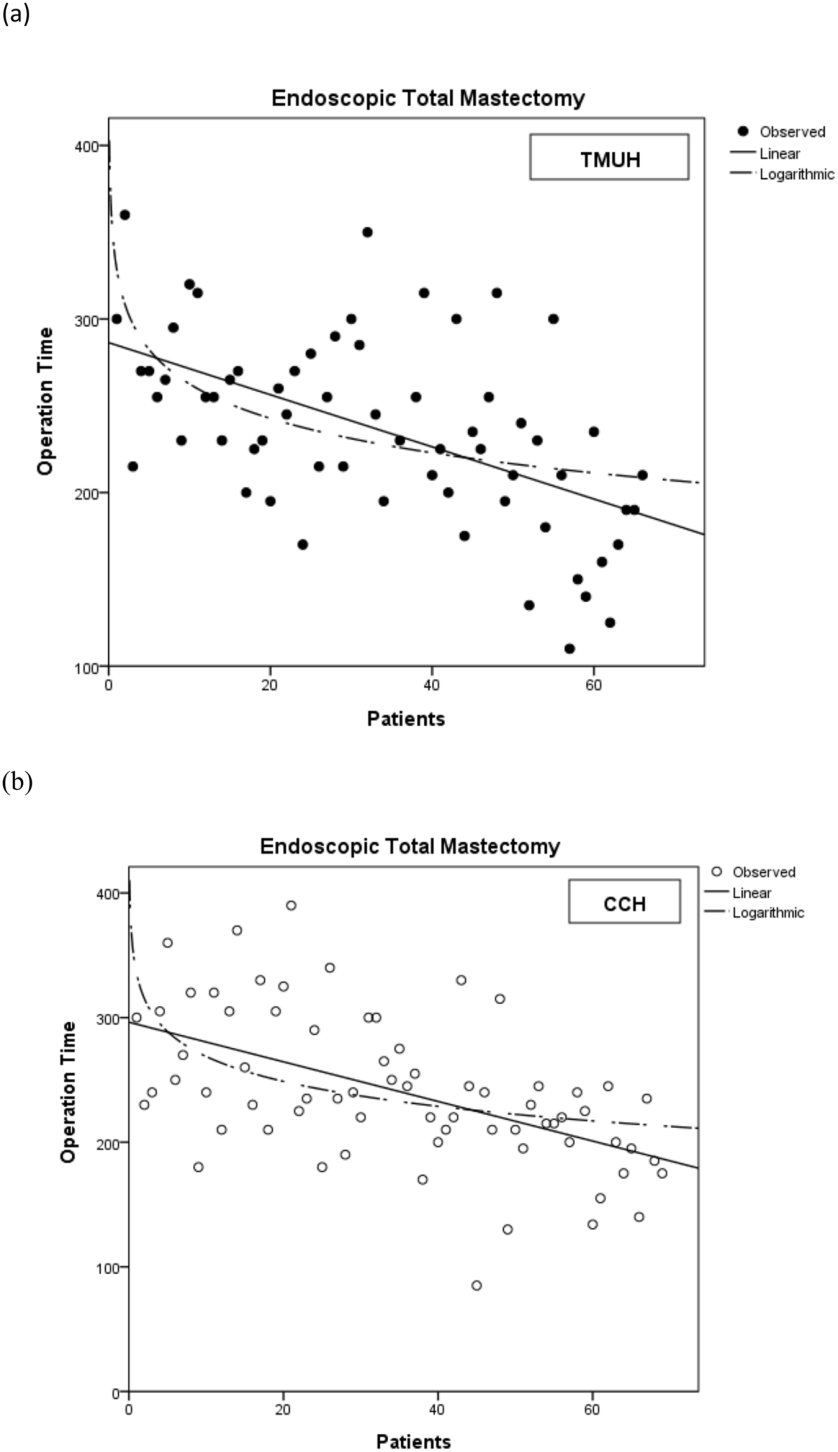
The learning curve of endoscopic total mastectomy. Both hospitals (a) TMUH and (b) CCH have the similar learning curve.

**Fig 3:**
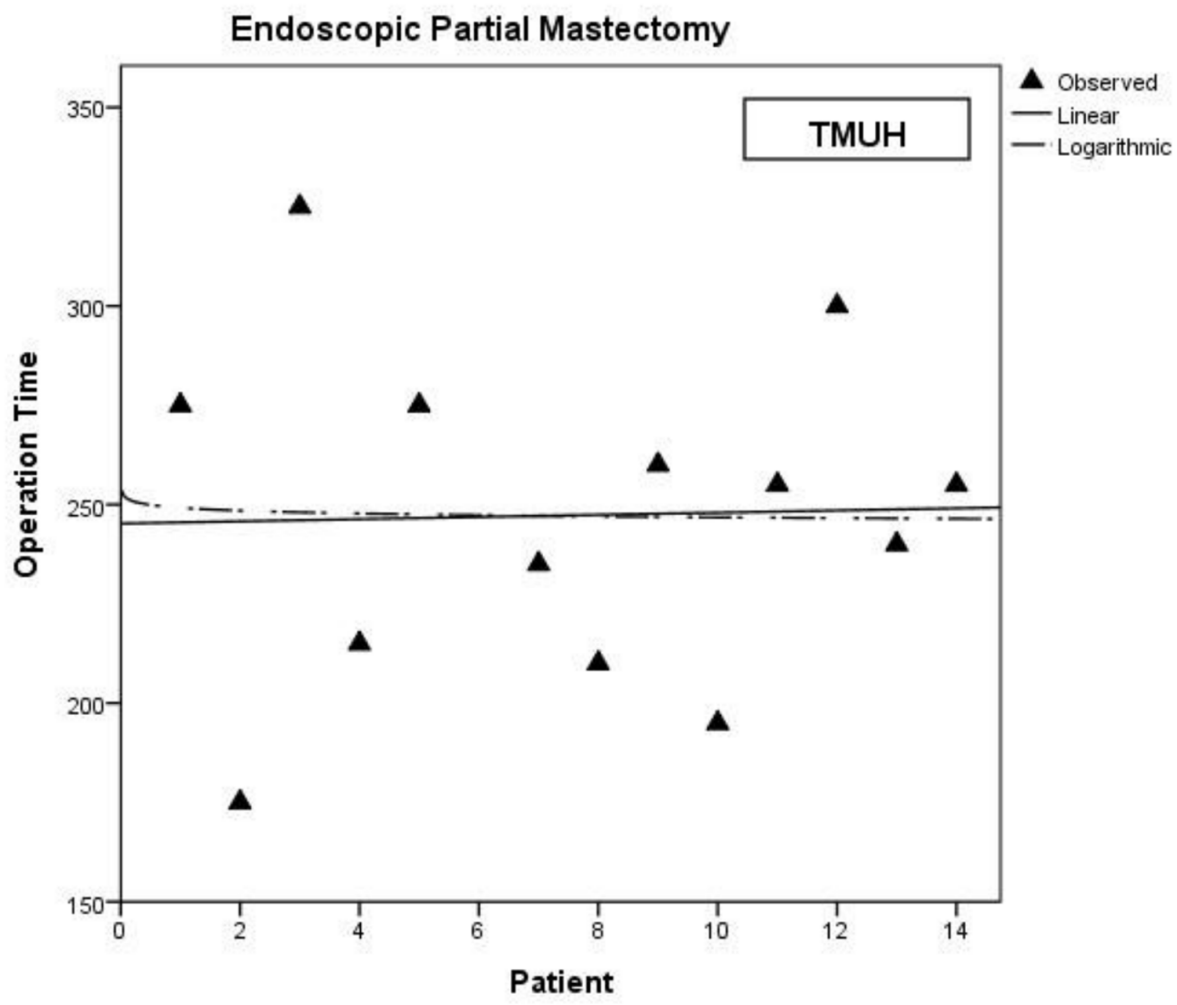
The learning curve of endoscopic partial mastectomy in TMUH.

**Fig 4:**
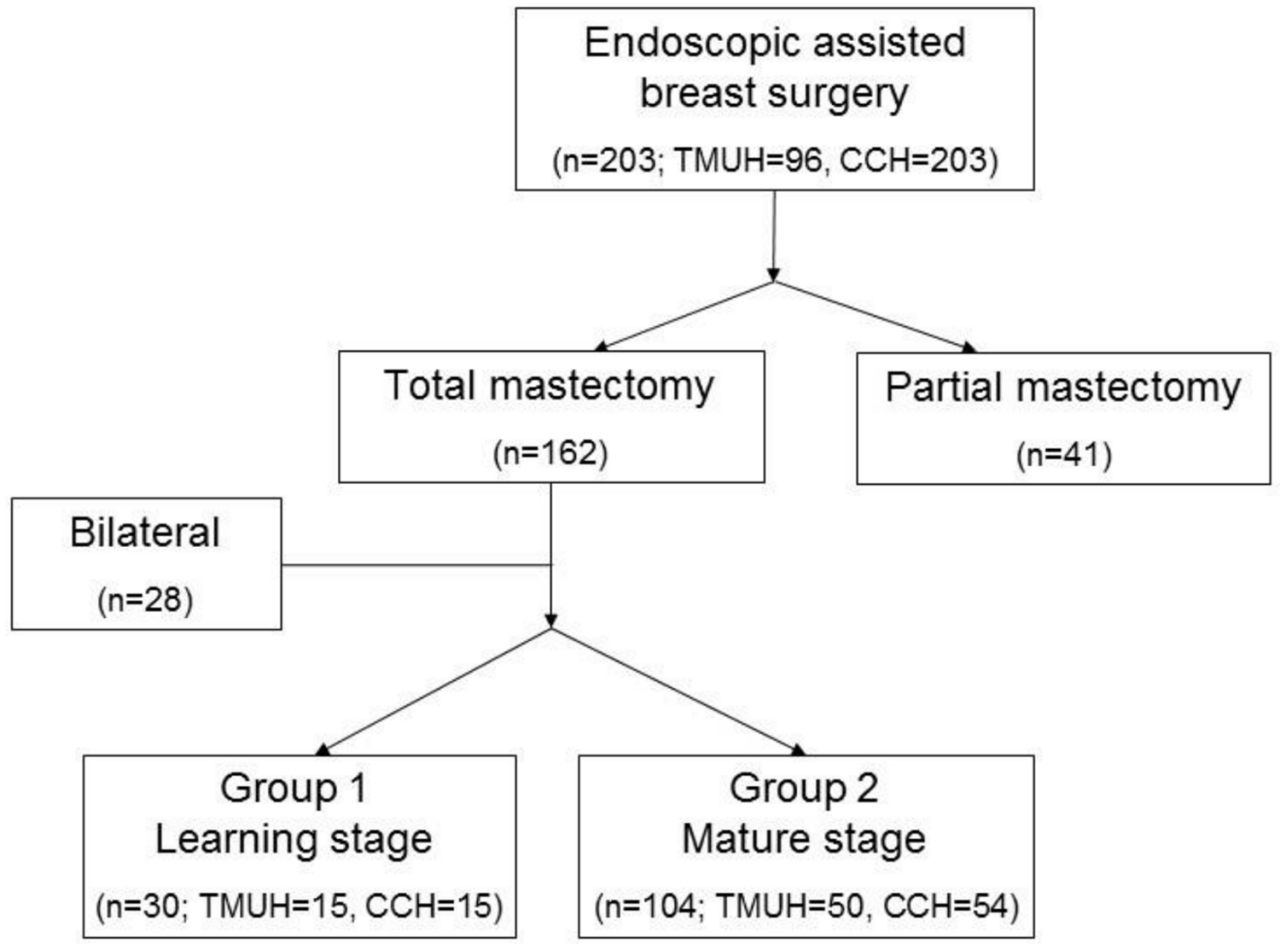
Learning group and mature group. Total 203 patients received an endoscopic mastectomy and 134 unilateral total mastectomy patients were divided into learning group and mature group.

Unilateral total mastectomy patients were enrolled for further analysis and a learning curve was identified in each hospital (Fig 2a and 2b). The inflection point occurred after approximately 15–17 patients. The surgeries were then further divided into a learning group, containing the first 15 patients, and a mature group, comprising the remaining patients. Patients who received partial mastectomies were also analyzed, since there was no obvious learning period in the endoscopic method for these surgeries (Fig 3). Bilateral mastectomy patients, most of whom underwent surgery on both breasts concurrently, were excluded, since this could affect the analysis results (Fig 4). A total of 134 patients were analyzed. Patient data, such as cancer stage, tumor size, and resected volume, were recorded, and operative variables were determined by retrospective chart review. Variables were compared using the χ^2^ test and Student’s *t*-tests. The study was approved by the Institutional Review Board of Changhua Christian Hospital (CCH IRB No.: 141224).

## Results

From 2011 to 2013, 134 patients were recruited for this study of the learning curve of endoscopic mastectomy. Their average age was around 50 years, and most of them were early breast cancer patients (stage 0: 27/134, 20.15%; stage I: 43/134, 32.09%; stage II: 55/134, 41.04%). There were no differences in patients’ basic data, tumor size, or cancer characteristics between the learning group and the mature group (Table 1). There were also no demographic differences in perioperative variables, including lymph node dissection surgery and reconstruction method (Table 2). Retro-areolar biopsy was routinely performed during the operation, and most patients (87/134, 64.93%) received a nipple-sparing mastectomy after a frozen section procedure showed that the cancer did not affect the nipple.

**Table 1:** Clinicopathologic factors according to operative experience.

	Learning phase (n = 30)	Mature phase (n = 104)	P
**Basic characteristics**			
Age	51.43 ± 7.98	49.81 ± 10.70	0.442
Height	156.26 ± 4.41	157.40 ± 6.04	0.343
Weight	55.99 ± 7.28	58.11 ± 9.88	0.282
BMI	22.88 ± 2.43	23.45 ± 3.69	0.435
**Tumor size (%)**			
Tis	6 (20%)	20 (19.2%)	0.35
T1	8 (26.7%)	42 (40.4%)	
T2	12 (40%)	34 (32.7%)	
T3	4 (13.3%)	6 (5.8%)	
**Lymph node (%)**			
N0	23 (76.7%)	77 (74.0%)	0.582
N1	4 (13.3%)	21 (20.2%)	
N2	3 (10.0%)	5 (4.8%)	
N3	0	1 (1.0%)	
**Stage (%)**			
0	6 (20.0%)	21 (20.2%)	0.087
I	6 (20.0%)	37 (35.6%)	
IIA	11 (36.7%)	23 (22.1%)	
IIB	3 (10.0%)	18 (17.3%)	
IIIA	4 (13.3%)	3 (2.9%)	
IIIB	0	0	
IIIC	0	1 (1.0%)	
**Nipple preservation (%)**			
Nipple sparing	18 (60.0%)	69 (66.3%)	0.521
Skin sparing (nipple not preserved)	12 (40.0%)	35 (33.7%)	
**Operation side (%)**			
Right	15 (50.0%)	42 (40.4%)	0.348
Left	15 (50.0%)	62 (59.6%)	
**Pathology (%)**			
Ductal carcinoma in situ	6 (20.0%)	21 (20.2%)	0.268
Invasive ductal carcinoma	16 (53.3%)	62 (59.6%)	
others	8 (26.7%)	21 (20.2%)	
**Neoadjuvant chemotherapy (%)**			
No	27 (90.0%)	98 (94.2%)	0.423
Yes	3 (10.0%)	6 (5.8%)	
**ER (%)**			
Negative	7 (23.3%)	21 (20.2%)	0.728
Positive	23 (76.7%)	82 (78.9%)	
**PR (%)**			
Negative	13 (43.3%)	39 (37.5%)	0.589
Positive	17 (56.7%)	64 (61.5%)	
**Her-2 (%)**			
-ive	23 (76.7%)	83 (79.8%)	0.639
+ive	7 (23.3%)	20 (19.2%)	

**Table 2:** The relationship between clinical factors and the operative experience.

	Learning phase (n = 30)	Mature phase (n = 104)	p
**Breast weight**	330 ± 141.69	337 ± 148.87	0.853
**Operation time (min)**	275.33 ± 46.35	228.91 ± 54.32	0.000*
**Local recurrence (%)**			
No	28 (93.3%)	103 (99.0%)	0.094
Yes	2 (6.7%)	1 (1.0%)	
**Lymph node surgery (%)**			
Sentinel	21 (70.0%)	74 (71.2%)	0.697
Sentinel then dissection	6 (20.0%)	24 (23.1%)	
dissection	3 (10.0%)	6 (5.7%)	
**Reconstruction (%)**			
No	13 (43.3%)	40 (38.5%)	0.889
Gel implant	14 (46.7%)	47 (45.2%)	
TRAM	1 (3.3%)	8 (7.7%)	
Others	2 (6.7%)	9 (8.6%)	
**Complication (%)**			
No	16 (53.3%)	83 (79.8%)	0.007*
Yes	11 (36.7%)	16 (15.4%)	
Not record	3 (10.0%)	5 (4.8%)	

*p < 0.05 means statistically significant

However, we found that there was a significant difference in operation time (275.3 vs. 228.9 min, p < 0.001) between the 30 patients in the learning group and the 104 patients in the mature group. The mature group spent less time in resection, but the weight of the resected breast tissue was not less than in the learning group (337 g vs. 330 g, p = 0.853). This shows that the learning curve was the result of the maturing of the surgical technique and not of a patient-selection bias. The surgical technique reached the mature phase after the first 15 operations.

Approximately 56.7% (17/30) of the patients in the learning group and 61.5% (64/104) of those in the mature group received immediate reconstructions. The most common reconstruction method was gel implant replacement, although nine patients received transverse rectus abdominis myocutaneous flap (TRAM) surgery instead. Post-operative complications included infection, poor wound healing, bleeding, prosthesis loss, and even nipple necrosis. The complication rate was significantly higher in the learning group.

## Discussion

Endoscopic breast surgery is a novel surgical method that uses a small incision wound to perform a complete resection of breast tissue. Many studies have shown that the oncological safety of endoscopic mastectomy is adequate relative to traditional operation methods [5, 7–9]. In our previous study from the Taiwan Endoscopic Breast Surgery Cooperative Group database, the recurrence rate was about 1% for patients who had received a unilateral total mastectomy, supporting the safety of endoscopic surgery for breast cancer [6].

Nipple-sparing mastectomies are another important issue. Most patients choose a nipple-sparing mastectomy for cosmetic reasons. However, breast surgeons are concerned about the residual duct in the nipple, which may increase the chance of local recurrence. According to similar studies on conventional nipple-sparing mastectomies, there is no significant difference in local recurrence when the nipple is preserved [8–11]. In our study, subareolar biopsies were routinely taken during the operation, and the decision on whether or not to preserve the nipple was made based on the results. In our study group, only one had local recurrence within the nipple post-surgery, and two others had local recurrence over the primary tumor site. The local recurrence rate was thus still low, even after nipple preservation.

A major belief concerning endoscopic breast surgery is that is it time consuming, since it usually takes more time than a traditional operation. For example, Kitamura et al. showed an increased operation time relative to open surgeries (237 ± 60 vs. 176 ± 32 minutes) [12]. Yamaguchi et al. compared endoscopic-assisted subcutaneous mastectomy (EASM) by the advanced skin flap method with EASM using a posterior approach. The surgical duration was 251 and 216 minutes, respectively [13]. In our study, an average of more than four hours was required to complete a one-sided mastectomy during the initial learning phase. Even after practicing the method, surgeons still spent an average of 228.9 minutes on a unilateral mastectomy (mature group). The combined data from the above two studies and ours show that endoscopy is indeed a time-consuming method, which exhibits limited improvement in this respect, even with mature skills.

The complication rate was one aspect that improved substantially from the learning to the mature phase. The post-operative complications of this procedure are quite different from those of traditional operations, because of skin and nipple preservation. The most common complication in nipple-sparing breast surgery is nipple ischemia, due to compromised blood supply from the periareolar incision [14], which also applies to endoscopic breast surgery. More details about the complications of EABS are given in our previous manuscript [6]. In that study, we found that the partial and total nipple ischemia rates were about 8.5% and 4%, respectively. In our experience, most of the ischemic change occurred 2–3 days after surgery and resulted in skin scaling, ulcers, or pigmentation changes. However, only a few patients developed nipple necrosis and had to receive further surgery. Not unexpectedly, another common complication was TRAM partial necrosis. In those surgeries, the residual complications included poor wound healing, hematoma, and infection. In this study, we have further clarified the reasons for these complications and can confirm that most of the complications occurred during the learning phase. Overall, the complication rate dropped dramatically, to one third of the initial rate, in the mature phase.

Endoscopic mastectomy is thus a time-consuming method that seems to have a higher complication rate than traditional methods. However, it improves cosmetic results, which was the primary reason for introducing it to Taiwan. This method can reduce the psychological trauma following mastectomy, especially if combined with immediate reconstruction. There are many studies demonstrating good cosmetic results after reconstruction [4, 12, 15–19]. In our study, around 60% of patients in both groups received immediate reconstructions, including a tissue expander, gel implant, or saline implant insertion.

In this retrospective review, we found no obvious learning curve in partial mastectomies. Endoscopic partial mastectomy may thus only differ slightly from conventional partial mastectomies. It is not difficult to shift from traditional to endoscopic surgery in such breast cancer cases. The difference in operation time seems to depend on patient-specific factors, rather than on the number of surgeries a surgeon has performed. For total mastectomies, a similar trend was observed at different hospitals. There was a learning curve and an inflection point after approximately 15 operations for each surgeon, regardless of where the surgeon was based. The difference in operation time depended on the number of endoscopic surgery procedures the surgeon had performed.

The endoscopic mastectomy process was modified during the three years of the study period. The main difference was the location at which the tissue was retrieved. From 2011 to 2012, all breast tissue was removed through the periareolar incision. In 2013, there was a gradual change, moving the retrieval site to the axillary wound. After 2014, the retrieval site for nearly all patients was the axillary wound. There are two reasons for this change. One is the necessity for axillary lymph node dissection in patients with lymph node metastasis. Since an axillary wound cannot be avoided in such cases, it is also used to remove the breast tissue. The other reason is that a smaller incision over the periareolar area may prevent ischemia or necrosis of the nipple-areolar complex. Minimizing the incision size of the periareolar wound may be one way to decrease the complication rate. However, our analysis showed no significant difference between the operation times of these two approaches.

### Study limitations

There are some factors that may influence the results of this study, such as the proficiency of team members. For example, the nurses and resident doctors were not initially familiar with the preoperative preparation or overall surgical procedures. There may thus have been more time spent in the learning phase due to variation in individual health care practitioners. However, we analyzed the results from two separate hospitals and observed a similar learning curve for both. This suggests that all breast surgeons can become competent in performing endoscopic mastectomies after practicing on a minimum of 15 cases.

## Conclusion

Our study is the first to discuss the learning curve of endoscopic total mastectomies. We found that this surgical method is innovative and that surgeons can perform it more quickly after an initial learning phase. Although it is still more time-consuming than traditional methods, it has advantages in improving cosmetic results and reducing the psychological impact on patients, if combined with immediate reconstruction.
