# Correction: The learning curve of endoscopic total mastectomy in Taiwan: A multi-center study

**DOI:** 10.1371/journal.pone.0183638

**Published:** 2017-08-17

**Authors:** Chin-Sheng Hung, Sheng-Wei Chang, Li-Min Liao, Cheng-Chiao Huang, Shih-Hsin Tu, Shou-Tung Chen, Dar-Ren Chen, Shou-Jen Kuo, Hung-Wen Lai, Ting-Mao Chou, Yao-Lung Kuo

There is an error in Fig 4. The case number of CCH is 107, not 203. Please see the complete, correct Fig 4 here.

**Fig 4:**
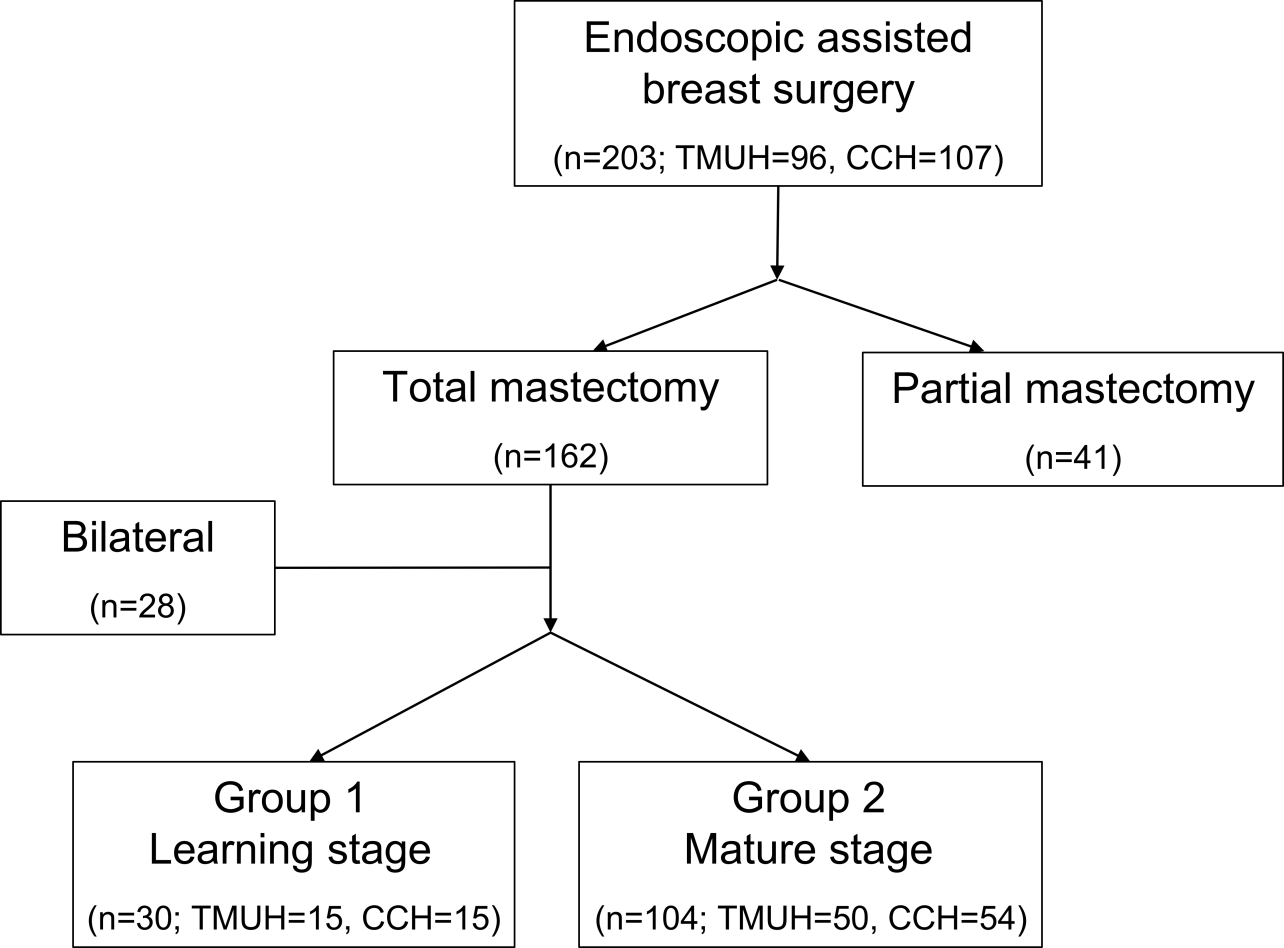
Learning group and mature group. Total 203 patients received an endoscopic mastectomy and 134 unilateral total mastectomy patients were divided into learning group and mature group.

## References

[1] Hung C-S, Chang S-W, Liao L-M, Huang C-C, Tu S-H, Chen S-T, et al (2017) The learning curve of endoscopic total mastectomy in Taiwan: A multi-center study. PLoS ONE 12(6): e0178251 https://doi.org/10.1371/journal.pone.0178251. doi: 10.1371/journal.pone.0178251 28594922 10.1371/journal.pone.0178251 PMC5464537

